# A Tailored App for the Self-management of Musculoskeletal Conditions: Evidencing a Logic Model of Behavior Change

**DOI:** 10.2196/32669

**Published:** 2022-03-08

**Authors:** Alice Berry, Carey McClellan, Ben Wanless, Nicola Walsh

**Affiliations:** 1 University of the West of England Bristol United Kingdom; 2 getUBetter Bristol United Kingdom; 3 Musculoskeletal Department St George’s University Hospitals National Health Service Foundation Trust London United Kingdom

**Keywords:** musculoskeletal, supported self-management, behavior change, digital health intervention, behavior change wheel

## Abstract

**Background:**

Musculoskeletal conditions such as joint pain are a growing problem, affecting 18.8 million people in the United Kingdom. Digital health interventions (DHIs) are a potentially effective way of delivering information and supporting self-management. It is vital that the development of such interventions is transparent and can illustrate how individual components work, how they link back to the theoretical constructs they are attempting to change, and how this might influence outcomes. getUBetter is a DHI developed to address the lack of personalized, supported self-management tools available to patients with musculoskeletal conditions by providing knowledge, skills, and confidence to navigate through a self-management journey.

**Objective:**

The aim of this study was to map a logic model of behavior change for getUBetter to illustrate how the content and functionality of the DHI are aligned with recognized behavioral theory, effective behavior change techniques, and clinical guidelines.

**Methods:**

A range of behavior change models and frameworks were used, including the behavior change wheel and persuasive systems design framework, to map the logic model of behavior change underpinning getUBetter. The three main stages included understanding the behavior the intervention is attempting to change, identifying which elements of the intervention might bring about the desired change in behavior, and describing intervention content and how this can be optimally implemented.

**Results:**

The content was mapped to 25 behavior change techniques, including information about health consequences, instruction on how to perform a behavior, reducing negative emotions, and verbal persuasion about capability. Mapping to the persuasive system design framework illustrated the use of a number of persuasive design principles, including tailoring, personalization, simulation, and reminders.

**Conclusions:**

This process enabled the proposed mechanisms of action and theoretical foundations of getUBetter to be comprehensively described, highlighting the key techniques used to support patients to self-manage their condition. These findings provide guidance for the ongoing evaluation of the effectiveness (including quality of engagement) of the intervention and highlight areas that might be strengthened in future iterations.

## Introduction

### Background

Musculoskeletal conditions such as joint pain are a growing problem, affecting 18.8 million people in the United Kingdom, which equates to 22% of the total burden of ill health across the population [1]. The importance of supporting people with musculoskeletal conditions in self-managing their own condition is increasingly acknowledged as a valuable tool for improving symptoms and facilitating healthy behaviors [2].

Self-management includes all the actions taken by people to recognize, treat, and manage their own health care independently of, or in partnership with, the health care system [3]. Supported self-management requires a whole systems approach that should be fully integrated into clinical care pathways, providing information and encouragement to help people gain more control over symptoms by understanding their condition through monitoring and taking appropriate action [4].

Adopting digital methods to support the self-management of a number of health conditions has become increasingly popular, gaining further traction during the COVID-19 pandemic with a rise in the number of digital health consultations [5]. Recent literature has highlighted the benefit of using digital technology in general practice, suggesting that the National Health Service (NHS) continues to invest in digital-first primary care [6].

Despite the rise in popularity, it is recognized that most digital health interventions (DHIs) are not well-described [7]. It is vital that the development of DHIs is transparent and can illustrate how individual components work, how they link back to the theoretical constructs they are attempting to change, and how this might influence outcomes [8]. During DHI development, it is important to clearly document techniques used by mapping content against recognized behavior change taxonomies [7]. This approach can allow for better implementation into practice, producing more effective and longer term engagement with healthy behaviors [9]. The National Institute for Health and Care Excellence (NICE) Evidence Standards Framework for digital health technology (DHT) also highlights the importance of using recognized effective behavior change techniques (BCTs), recommending that evidence is available to illustrate how BCTs within an intervention are consistent with recognized behavior change theory and recommended practice (aligned with guidance from NICE or relevant professional organizations) and appropriate for the target population [10]**.**

As part of the Small Business Research Initiative Health Care and an NHS England program to bring new technologies to the NHS, getUBetter was contracted to develop, deploy, evaluate, and optimize its DHT to scale into the NHS. The contract was titled *Integrated Self-management and Prevention for Multiple Musculoskeletal Conditions and Pathways*. getUBetter worked with teams from the Wandsworth NHS Clinical Commissioning Group, St Georges University Hospitals NHS Foundation Trust, the Digital Evaluation Unit at the Health Innovation Network in South London, and the University of the West of England in Bristol to evaluate the DHI in a real-world setting. Part of this evaluation focused on evidencing the underpinning logic model of behavior change in the DHI; this element of the project is reported in this paper.

getUBetter [11] was developed to address the recognized problem within the health system of a lack of personalized, supported self-management tools available to patients with musculoskeletal conditions that provide the knowledge required to trust in, and successfully navigate through, a self-management journey.

The aim of the getUBetter self-management app is to empower individuals to self-manage from the start of an acute phase of an injury or episode to recovery and long-term condition management. It intends to do this by providing knowledge, developing skills, identifying and developing support strategies, and using persuasive language to develop positive beliefs about one’s self-management capability. Patients are prescribed getUBetter by a clinician (or practice staff) face to face, virtually, or via an SMS text message as part of routine care. Patients can also self-refer from a health care provider’s website. Musculoskeletal conditions include back, back and leg, neck, shoulder, knee, ankle, and lower limb soft injury, and patients can be linked to ≥1 local injury or condition pathway simultaneously, such as lower back pain and new ankle injury.

getUBetter was designed for both new and recurrent conditions and is specifically tailored to local care pathways, supporting triage, day-to-day recovery, referral when needed, connection to local rehabilitation programs, and prevention and well-being when a patient is back to their normal.

The getUBetter platform *houses* standardized national care pathways for the range of conditions based on evidence and national clinical treatment guidelines. Before the getUBetter platform and app are integrated into a health care system (eg, into general practitioner practices), urgent care centers, or physiotherapy departments, getUBetter works with the local clinical teams to configure each element of the local care pathway. There are three pillars of configurability: the day-by-day condition (lived experience of the patient), the end-to-end clinical condition pathways, and the local health system (health care and social care and public health). Patients are then connected to their specifically tailored local care pathways via either prescriptions or self-referrals.

getUBetter adopts a whole pathway approach that includes a symptoms checker, use of stratification tools, and automated referrals. Targeted and personalized content aims to help patients understand their stage of recovery, providing knowledge to strengthen confidence; reduce negative behaviors; and include a recovery plan around medication, effective treatment options, and the ability to self-monitor recovery. Evidence-based advice is provided on a range of topics such as what to expect at different stages of recovery; information about pain relief; guidance on referral options; information about driving, work, and other everyday activities; a symptom checker; information about red flags; and a variety of exercises demonstrated by physiotherapists are available to guide patients through their recovery journey (for more examples, see Multimedia Appendix 1 [12]).

The co-design process included patient and public involvement groups from the local clinical commissioning group, general practitioners, physiotherapists of all grades, consultant or advanced practice physiotherapists for most conditions, carers, charities such as Age UK, and patients. Patients from the patient and public involvement group were a broad group with differing technical capabilities, and the focus groups comprised patients of all ages. Importantly, ongoing user experience was captured on a regular basis during development.

getUBetter is suitable for all patients aged ≥18 years. The platform and app have been co-designed with all stakeholder groups to minimize digital exclusion; for example, easy-to-use buttons, text size to 200%, and an easy-to-access web version. getUBetter works closely with organizations, patients, and all stakeholders to minimize digital exclusion and support *care as usual* and actively seeks to support patients who may be having difficulties accessing the solution.

### Aim of Study

The aim of this study was to develop a logic model of behavior change for getUBetter, illustrating how the content and functionality of the app are aligned with recognized behavioral theory, effective techniques, and recommended clinical practice. This paper describes the development of a behavior change model for getUBetter using widely used models and frameworks described in more detail in the following sections.

## Methods

### Overview

The behavior change wheel (BCW) [13], Capability Opportunity Motivation-Behavior (COM-B) model [14], and BCT Taxonomy (BCTT) Version 1 (BCTTv1) [7] were used to map the BCTs present and the behavior change model for getUBetter. The persuasive systems design (PSD) framework [15] was used to describe the functionality and delivery of content.

To replicate and implement behavior change interventions in practice, we need an agreed-upon language to report their content or *active ingredients*. There are a range of tools that can be used to do this, and one that is widely used is the BCW, which provides a systematic way of characterizing interventions. It is often used in conjunction with the COM-B framework [14], a theoretical framework that incorporates key components (capability, opportunity, and motivation) considered to affect behavior. The COM-B model is a starting point for understanding behavior in the context in which it occurs. The central tenet of the model is that for any behavior to occur (1) there must be the *capability* to do it (ie, physical strength, knowledge, skills, and stamina), (2) there must be the *opportunity* for the behaviors (ie, physically accessible, affordable, socially acceptable, and sufficient time), and (3) there must be sufficiently strong *motivation* [12]. BCTTv1 was developed to specify content in terms of BCTs, the smallest components of behavior change interventions that can bring about change [7]. BCTTv1 is a hierarchically structured taxonomy of 93 distinct BCTs, including labels, definitions, and examples.

Although the BCTT enables the illustration of BCTs adopted within an intervention, the additional inclusion of the PSD framework was important to demonstrate how BCTs were translated into practice and ensure that important aspects of functionality were adequately described. The PSD framework was developed to guide the process of designing and evaluating persuasive systems, which highlights 28 design principles for persuasive system content and functionality, categorized into four areas: primary task, dialog, system credibility, and social support categories [15].

The following sections describe the 3 stages that were conducted to illustrate the behavior change model for getUBetter. It is important to highlight that this process was nonlinear, and *mapping* procedures moved between stages iteratively to link BCTs with theory, intended outcomes, functions, and categories of the BCW, as well as persuasive system design elements.

### Stage 1: Understanding the Target Behavior

Several team meetings comprised clinicians, developers, user experience and user interface designers, project managers, and product owners from getUBetter alongside other clinicians from the stakeholder groups and researchers with experience in developing self-management and behavior change interventions from the University of the West of England. The clinicians included an advanced physiotherapy practitioner in primary care and emergency care and a consultant musculoskeletal physiotherapist. The meetings were held to understand the health problems that getUBetter aims to address and the behavior(s) that it attempts to change and describe what needs to change to achieve the intended outcome(s). This process enabled the aims and objectives of getUBetter to be comprehensively described and illustrated in a logic model of the problem.

Next, the COM-B model and the Theory and Technique tool [16] were used to link the BCTs present in getUBetter to relevant theoretical domains. The Theory and Technique tool links the theoretical domains framework [17] to the COM-B model to provide a more granular understanding of how constructs are associated with capability, opportunity, and motivation (eg, the theoretical domain *beliefs about capabilities* is linked to *reflective motivation* within the COM-B model). This step highlighted to the team the potential areas where getUBetter might be strengthened by adding in BCTs commonly reported in similar published literature but which were not currently present in getUBetter.

### Stage 2: Mapping Intervention Options

**Figure 1:**
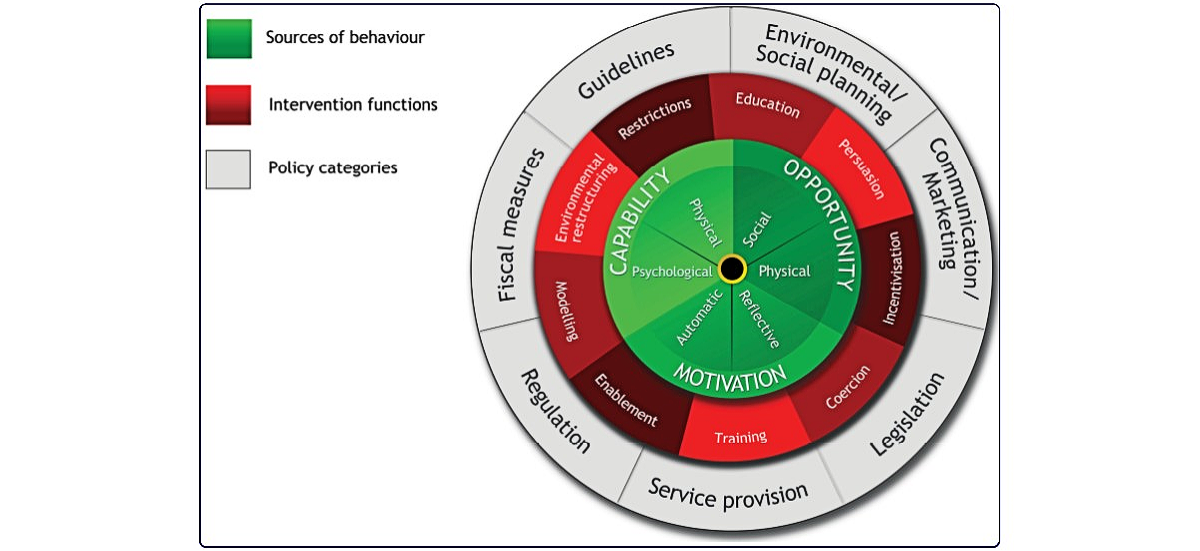
Behavior change wheel [13].

This stage involved linking information gathered previously in stage 1 (the *behavioral diagnosis*) to the intervention functions (Figure 1) and policy categories (Figure 1) through which getUBetter is currently being implemented. Team meetings were held to agree on the links between the BCTs present in getUBetter and relevant implementation functions and policy categories. Once again, this was an opportunity to explore the areas that could be strengthened in future iterations.

### Stage 3: Mapping Content and Implementation Options

**Figure 2:**
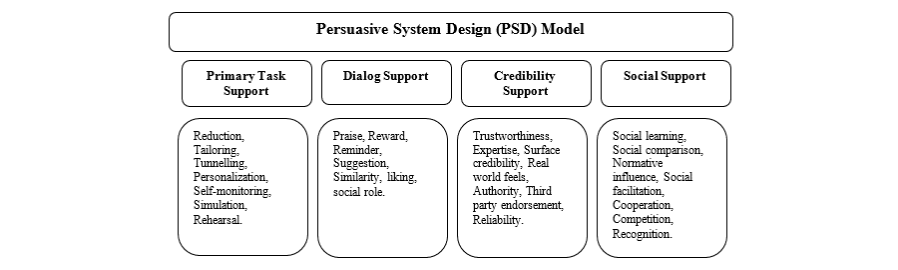
Persuasive systems design model.

This final stage involved using the BCTT [7] to identify and map BCTs present within getUBetter and the PSD model to illustrate how the BCTs were being delivered (Figure 2).

The results from the abovementioned stages described were then consolidated into a logic model of behavior change for getUBetter.

## Results

### Overview

An initial mapping exercise was completed during November 2019 and April 2020 by the lead author (AB), who discussed and agreed on the mapping with team members. The mapping process was nonlinear and iterative, moving between the stages described earlier and guided by team discussions. This initial mapping exercise enabled the team to identify particular areas of the app that could be strengthened by adding specific BCTs commonly used in other interventions [12] but which were not currently present in getUBetter. Following ongoing iterative design and development of getUBetter throughout 2020, a second mapping exercise took place between January 2021 and March 2021 to highlight additional techniques and functionalities.

The following sections describe each stage of *mapping round 1* in more detail and the additional techniques and changes in functionality highlighted during *mapping* *round 2*.

### Stage 1: Understanding the Target Behavior

#### Overview

The aim of getUBetter is to empower individuals to self-manage their musculoskeletal condition by providing support and advice throughout their recovery journey and prevent future episodes. Objectives were clustered into three subgroups: knowledge and skills, supportive environment, and motivation and self-efficacy. The aims and objectives are described in more detail in the following sections.

#### Behavioral Aims and Objectives of getUBetter (version 2.0)

##### Knowledge and Skills

These objectives aim to provide the patient with sufficient knowledge about their musculoskeletal condition, enabling them to develop sufficient skills to conduct positive self-management behaviors.

The objectives were as follows:

1. Educate about normal physiological responses (ie, symptoms and time frames)
2. Train the patient in personally relevant skills (such as reducing sedentary behavior, introducing graded tasks, and relevant exercises) to enable them to manage symptoms and implement positive self-management behaviors

##### Supportive Environment

These objectives aim to train the patient to identify and develop supportive aspects of physical and social environments.

The objectives were as follows:

1. Guide understanding of the interpersonal influences on self-managing their musculoskeletal condition and how the environment can be restructured to provide optimal support
2. Promote reflection on environmental and emotional aspects affecting self-management (time, resource use, and place and well-being)
3. Highlight opportunities for restructuring the physical and social environments to best support self-management behaviors
4. Enable patient to navigate the health system and understand treatment pathways and referral options

##### Motivation and Self-efficacy

These objectives aim to enable the patient to form positive beliefs and feelings toward self-managing their musculoskeletal condition and support the formation of a personally relevant action plan for self-management behaviors

The objectives were as follows:

1. Persuade the patient about their capability and enable them to perform self-management behaviors such as action planning or healthy lifestyles
2. Educate (to strengthen beliefs) about the positive consequences (outcomes) of the directed self-management behaviors
3. Train the patient to form clear action plans for conducting self-management behaviors and self-monitor behaviors and outcomes
4. Provide prompts and cues to encourage self-management behaviors.

**Figure 3:**
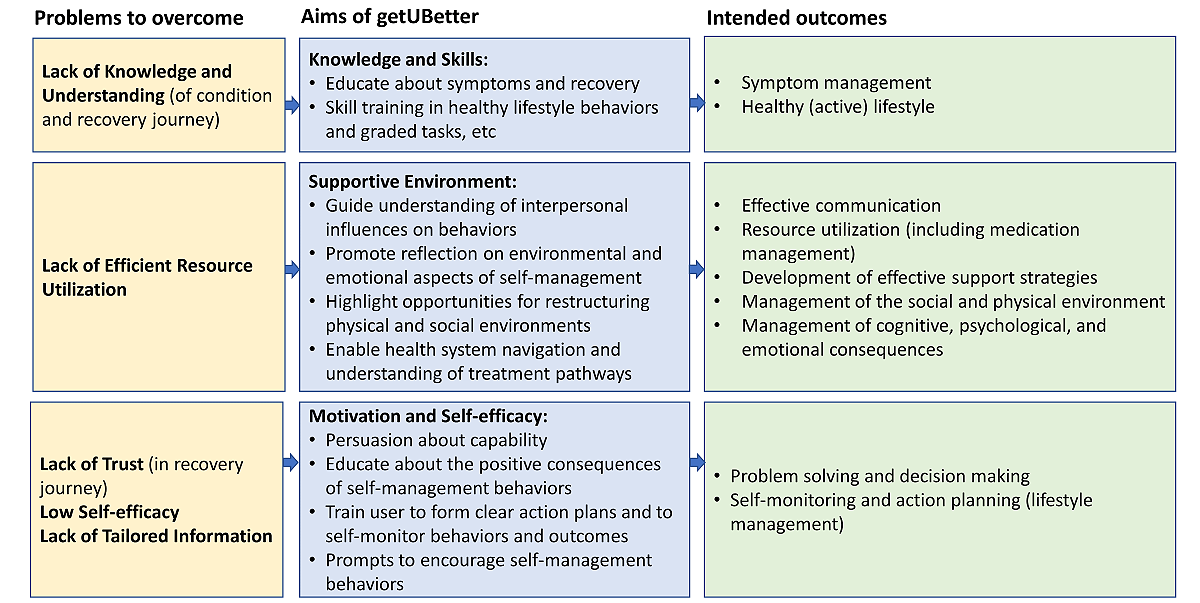
Logic model of problem.

Specific self-management behaviors were also identified (intended outcomes; Figure 3). Figure 3 shows the logic model of the problem(s) that getUBetter aims to address, how it aims to engage patients with positive self-management behaviors, and the intended outcomes.

The next step was to identify how BCTs present in getUBetter were linked back to the theoretical constructs they were attempting to change (using the BCW guidance and the Technique and Theory Tool see *Methods* section). Textbox 1 illustrates these links.

This stage highlighted a number of BCTs that could be beneficial to include in future iterations of getUBetter. More specifically, BCW guidance highlighted that BCTs related to social comparison (5.2) and goal setting and monitoring (ie, 1.1, 1.5, and 2.3) were reported to have been used in previous behavior change interventions [12].

**Capability, Opportunity, and Motivation–Behavior analysis (coded to Behavior Change Technique Taxonomy version 1).**

**Capability**

- Knowledge and skills
  4.1 Instruction on how to perform a behavior
  5.1 Information about health consequences
  8.1 Behavioral practice and rehearsal
  8.7 Graded tasks
- Memory, attention, and decision processes
  7.1 Prompts and cues
- Behavioral regulation
  1.2 Problem solving
  8.2 Behavior substitution
  11.2 Reduce negative emotions

**Opportunity**

- Environmental context and resources
  7.1 Prompts and cues
  12.1 Restructuring the physical environment
  12.2 Restructuring the social environment
- Social influences and identity
  3.1 Social support (unspecified)
  6.2 Social comparison
  9.1 Credible source

**Motivation**

- Beliefs about capabilities
  1.2 Problem solving
  4.1 Instruction on how to perform a behavior
  6.1 Demonstration of the behavior
  8.1 Behavioral practice and rehearsal
  8.7 Graded tasks
  15.1 Verbal persuasion about capability
- Beliefs about consequences
  5.1 Information about health consequences
  5.6 Information about emotional consequences
- Intentions
  5.1 Information about health consequences
- Reinforcement
  7.1 Prompts and cues
- Emotion
  11.2 Reduce negative emotions

### Behavioral Support at Different Stages of Recovery

**Figure 4:**
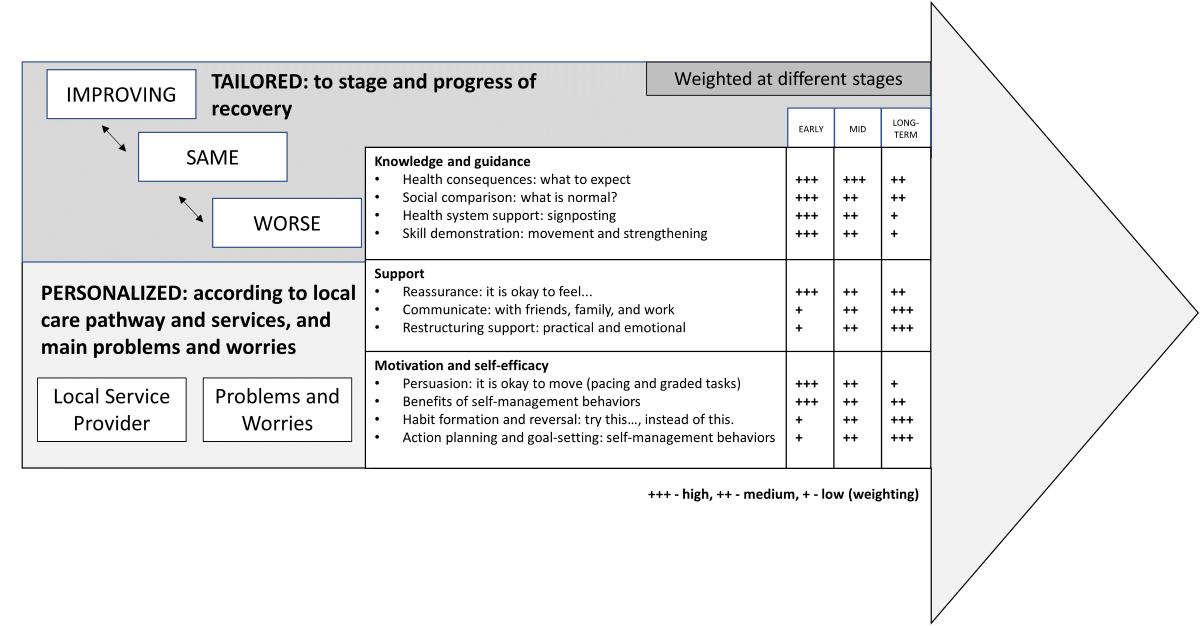
Support at different stages of recovery.

The mapping process described in this paper provided an opportunity for the team to highlight that a key aim of getUBetter is to empower individuals to self-manage their condition wherever they may be on their recovery journey. This issue was not governed by the mapping process; however, the team agreed that it was important to describe this during the mapping process to illustrate the nuance of delivering BCTs in both a personalized and tailored way. The team also consulted with clinicians at this point to gather opinions on the potential weighting of BCTs at different stages of recovery. Although there were some differing opinions, there was general agreement in line with the weighting shown in Figure 4.

Figure 4 illustrates how, within getUBetter, content is tailored to two main variables: (1) timeline of the recovery journey, moving through early-term, mid-term, and long-term stages of recovery, and (2) status—how the patient is progressing at any given time point (ie, getting better, staying the same, or getting worse). Content is also personalized to two main variables: (1) an individual’s local care pathway, local services, and health care provider and (2) patients identifying their main problems and worries to receive personalized advice. Content automatically adjusts to the stage of the recovery journey and individual progress, and this targeted content helps the patient to understand their stage of recovery while providing personalized support. Figure 4 illustrates how content is both tailored and personalized.

The full range of BCTs is present across all stages of recovery, although weighted differently according to individual progress and stage of recovery. For example, in the earlier stages of recovery, the key focus is on reassurance and providing knowledge about what to expect, what is normal, and having the confidence to seek help if needed. Later on, the focus might shift to making an action plan and considering healthy lifestyle changes to support effective self-management (Figure 4 also illustrates how content is weighted at different stages of recovery). The main concerns and problems can also be identified to further personalize content to strengthen confidence, reduce negative behaviors, and provide ongoing reassurance and persuasion about conducting self-management behaviors.

### Stage 2: Mapping Intervention Options

BCTs present in getUBetter were then mapped to the intervention functions and policy categories of the BCW. This was another opportunity to evaluate how well the existing BCTs in getUBetter represented each intervention function.

Relevant intervention functions included education, persuasion, training, environmental restructuring, modeling, and enablement. Policy categories relevant to support the delivery of getUBetter included communication and marketing, guidelines, fiscal measures, regulation, service provision, and environmental and social planning. Multimedia Appendix 2 provides a detailed description of the links between the COM-B components and the intervention functions.

### Stage 3: Mapping Content and Implementation Options

#### Mapping: Round 1

##### BCT Mapping

During the first round of mapping, getUBetter was mapped to 22 BCTs in the BCTTv1 (Textbox 2).

**Mapping of behavior change techniques in getUBetter.**

**Behavior change technique (coded to Behavior Change Technique Taxonomy version 1)**

- 1.1 Goal setting (behavior; *added in round 2*)
- 1.2 Problem solving
- 1.5 Review behavior goal(s) (*added in round 2*)
- 2.3 Self-monitoring of behavior (*added in round 2*)
- 2.5 Monitoring of outcome(s) of behavior without feedback
- 3.1 Social support (unspecified)
- 3.3 Social support (emotional)
- 4.1 Instruction on how to perform a behavior
- 5.1 Information about health consequences
- 5.6 Information about emotional consequences
- 6.1 Demonstration of the behavior
- 6.2 Social comparison
- 7.1 Prompts and cues
- 8.1 Behavioral practice and rehearsal
- 8.2 Behavior substitution
- 8.3 Habit formation
- 8.4 Habit reversal
- 8.7 Graded tasks
- 9.1 Credible source
- 11.1 Pharmacological support
- 11.2 Reduce negative emotions
- 12.1 Restructuring the physical environment
- 12.2 Restructuring the social environment
- 12.6 Body changes
- 15.6 Verbal persuasion about capability

##### PSD Elements

Mapping to the PSD model illustrated the use of a number of persuasive design principles, including tailoring, personalization, simulation, and reminders, which were used to deliver the content of getUBetter.

Table 1 below shows how getUBetter incorporates key persuasive design principles from the PSD model into its functionality and content. In addition, it was recognized that some of these might be adapted and strengthened in future iterations.

**Table 1:** Persuasive system design elements in getUBetter.

Elements and descriptions				Existing content in getUBetter
**Primary task support**				
	**Reduction**			
		A system that reduces complex behavior into simple tasks helps patients perform the target behavior, and it may increase the benefit–cost ratio of a behavior.	Simplification of national guidelines and musculoskeletal pathway Simplifies the journey through local pathway and access to local services Automates referrals and promotes self-choice Writing style—translates complex messages into simple language Simplifies tasks—related to time point	
	**Tunneling**			
		Using the system to guide patients through a process or experience provides opportunities to persuade along the way.	Tunneled by timeline—content provided depending on choice: improving, staying the same, or getting worse Full autonomy for which order to visit app pages Symptom checker—red flags directing the patient to a health professional (lock screen)	
	**Tailoring**			
		Information provided by the system will be more persuasive if it is tailored to the potential needs, interests, personality, use context, or other factors relevant to a patient group.	All content tailored to the day of recovery and recovery status (getting better, staying the same, or getting worse) Feedback zone—tailored information around main worries and problems	
	**Personalization**			
		A system that offers personalized content or services has a greater capability for persuasion.	My main problems My main worries (provides tailored information) Personalized information to local services (local trust specific)	
	**Self-monitoring**			
		A system that keeps track of one’s own performance or status supports the patient in achieving goals.	Diary, calendar, and goal-setting functions are available in the app, which are linked to prompts and reminders	
	**Simulation**			
		Systems that provide simulations can persuade by enabling patients to observe the link between cause and effect immediately.	Videos about simple exercises and activities of daily living	
	**Rehearsal**			
		A system providing means with which to rehearse a behavior can enable people to change their attitudes or behavior in the real world.	Videos and instructions on how to perform a behavior—exercising specific and general day-to-day activities	
**Dialogue support**				
	**Praise**			
		The system should use praise via words, images, symbols, or sounds as a way of providing patient feedback information based on their behaviors.	Persuasive messaging throughout (including information about health consequences, reducing negative emotions, and verbal persuasion about the capability to perform self-management behaviors—covered in BCTTv1^a^ mapping)	
	**Reminders**			
		If a system reminds patients of their target behavior, the patients will more likely achieve their goals. The system should remind patients of their target behavior during the use of the system.	Automated reminders to log back into the app	
	**Suggestion**			
		The system should suggest that patients conduct behaviors during the system use process.	Covered in BCTTv1 mapping	
	**Liking**			
		A system that is visually attractive for its patients is likely to be more persuasive.	Ongoing design updates	
**System credibility support**				
	**Trustworthiness**			
		The system should provide information that is truthful, fair, and unbiased.	Follows national guidelines, NHS^b^ guidelines, and NICE^c^ digital health guidelines and is validated by CCG^d^—linked to the musculoskeletal pathway Local GP^e^ logo	
	**Expertise**			
		The system should provide information showing knowledge, experience, and competence.	Developed by experts	
	**Surface credibility**			
		The system should have a competent look and feel.	P-mat—tested with patients—high level of usability and acceptability No advertisements	
	**Real-world feel**			
		The system should provide information about the organization and actual people behind its content and services.	NHS badge, local services, local CCG—credible source In GP practices	
	**Authority**			
		The system should refer to people in the role of authority.	Links back to GP and other NHS services	
	**Third-party endorsements**			
		The system should provide endorsements from respected sources.	Links back to GP and other NHS services	
	**Verifiability**			
		The system should provide means of verifying the accuracy of the site content via outside sources.	Links back to GP and other NHS services	
**Social support**				
	**Social learning**			
		The system should provide means of observing other patients who are performing their target behaviors and seeing the outcomes of their behavior.	Some target behaviors were displayed in videos but not all No illustrations of outcomes of behavior (eg, positive stories of improvement)	

^a^BCTTv1: Behavior Change Technique Taxonomy Version 1.

^b^NHS: National Health Service.

^c^NICE: National Institute for Health and Care Excellence.

^d^CCG: clinical commissioning group.

^e^GP: general practitioner.

#### Mapping: Round 2

The first round of mapping highlighted the potential value of adding specific BCTs to strengthen it in future iterations. Following a number of changes during ongoing development, a second round of mapping was conducted, resulting in the identification of 3 additional BCTs (Textbox 2). This was translated to a number of areas within the app that were strengthened; for example, the addition of a goal-setting section linked to a calendar function where patients can log progress and self-monitor their recovery journey.

A number of PSD elements were also strengthened, including more direct links between sections, production of additional guidance and instruction videos, development of video strings to guide patients through videos, and addition of a traffic light system to guide the patient to the most appropriate exercises.

#### Logic Model of Behavior Change for getUBetter

**Figure 5:**
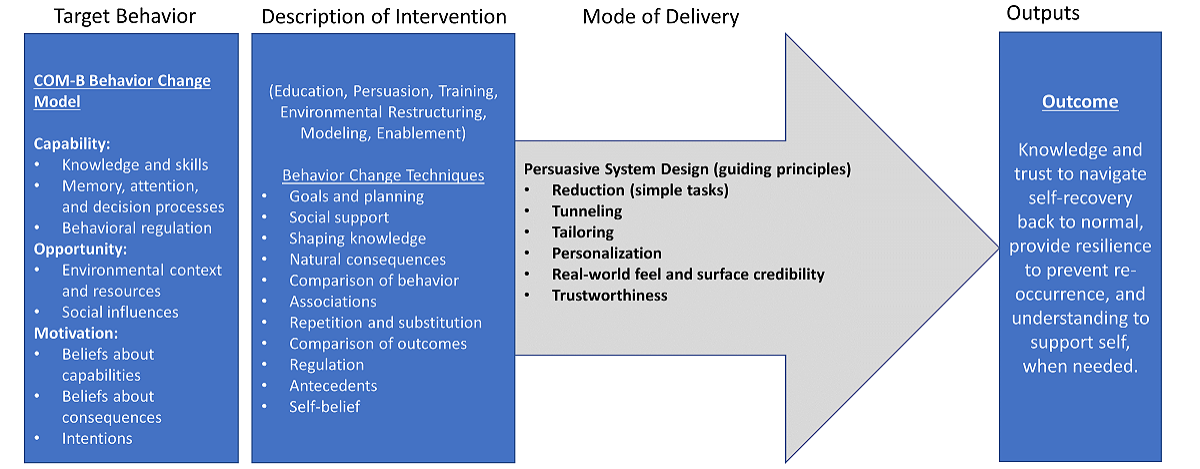
Logic model of behavior.

Figure 5 illustrates the full behavior change model for getUBetter. It provides details of the target COM-B elements that getUBetter intends to change (target behaviors) and describes the intervention functions and BCT groups that are used to make this change (description of intervention), the PSD elements present that deliver the BCTs (mode of delivery) via the app, and how the intervention as a whole affects the intended outcomes (outputs).

## Discussion

### Principal Findings

The aim of this project was to evidence the behavior change model underpinning getUBetter. A range of models and frameworks were adopted to demonstrate how the content and functionality of the intervention aligns with the recognized behavioral theory, effective BCTs, and recommended clinical practice.

This study offers a method for illustrating a behavior change model for a complex DHI and is in line with Medical Research Council complex intervention development guidelines [18], NICE evidence standards framework for DHT [10], and literature that has adopted similar methods to illustrate the development of behavior change interventions in musculoskeletal health [19]. It recognizes the value of using overlapping frameworks to capture recognized BCTs (using the BCTTv1) while highlighting the novel functionality of the intervention (using the PSD model), which importantly enables transparency and replicability.

The behavior change model can be seen as a blueprint for behavior change that can be used to guide future iterations of the intervention and the development of content for other musculoskeletal conditions, which are currently in development. The process also enabled the team to identify potential areas of the intervention that could be strengthened and highlighted the potential value of adding BCTs not present in the initial mapping round.

The mapping exercise opened up an opportunity for team discussion about the relative importance of different BCTs at different time points of recovery. getUBetter was developed so that content is tailored depending on where each individual is within their own recovery journey and whether symptoms are improving, staying the same, or getting worse. Future research should explore with patients the relative importance of different behavioral support at different stages of recovery to help identify if and how behavioral support can be more tailored and personalized in future iterations of the DHI. This is a novel area that is yet to be explored in the literature and requires further investigation.

### Limitations

Mapping and coding of BCTs are often open to interpretation, and we recognize the potential bias from the project team. However, a number of team meetings (which included clinicians with extensive experience in treating musculoskeletal conditions and academics with experience in developing behavior change interventions) were held to discuss the content and reach consensus when linking content to BCTs.

Coproduction and engagement with patients (a planned next step) would add value and help to learn more about potential areas that could be better represented and additional BCTs relevant and acceptable to the musculoskeletal population.

### Next Steps

Throughout this process, the importance of exploring acceptability (of intervention content) and usability (of the digital intervention) was recognized as a key factor in understanding how the intervention will be adopted and used by patients, and work in this area is being planned in future studies. The behavior change model described in this paper will act as a useful foundation for exploring these topics further with patients, and we recognize the important need to understand the characteristics and strategies that are most effective in promoting both the engagement with digital technologies [20] and the healthy behaviors that they intend to promote. Future work will also explore the effectiveness of this DHI on a larger scale.

### Conclusions

This process enabled the proposed mechanisms of action and theoretical foundations of a DHI to be comprehensively described, highlighting the key BCTs used to empower patients to self-manage their condition. These findings provide guidance for the ongoing evaluation of the effectiveness of the intervention.

The new standard framework for DHT includes the evaluation of behavior change [10]. There is limited evidence about how people engage with and act upon information delivered via digital behavior change interventions, especially in tools that span acute to long-term conditions. We have demonstrated that it is possible to evaluate the behavioral mechanisms of this DHT using a range of models and frameworks to illustrate a behavior change model for getUBetter.

## Appendix

Multimedia Appendix 1 Examples of APP pages and links to behavior change techniques.

Multimedia Appendix 2 getUBetter—linking the Capability, Opportunity, Motivation–Behavior (COM-B) components to intervention functions and policy categories from the behavior change wheel.

## Abbreviations

BCT: behavior change technique
BCTT: Behavior Change Technique Taxonomy
BCTTv1: Behavior Change Technique Taxonomy Version 1
BCW: behavior change wheel
COM-B: Capability, Opportunity, Motivation–Behavior
DHI: digital health intervention
DHT: digital health technology
NHS: National Health Service
NICE: National Institute for Health and Care Excellence
PSD: persuasive systems design
