# Supplementary material for: A Tailored App for the Self-management of Musculoskeletal Conditions: Evidencing a Logic Model of Behavior Change
Source: JMIR Form Res. 2022 Mar 8;6(3):e32669. doi: 10.2196/32669 (PMC8941434; doi:10.2196/32669)
Supplement: Multimedia Appendix 1 [file formative_v6i3e32669_app1.pptx]

## Slide 1
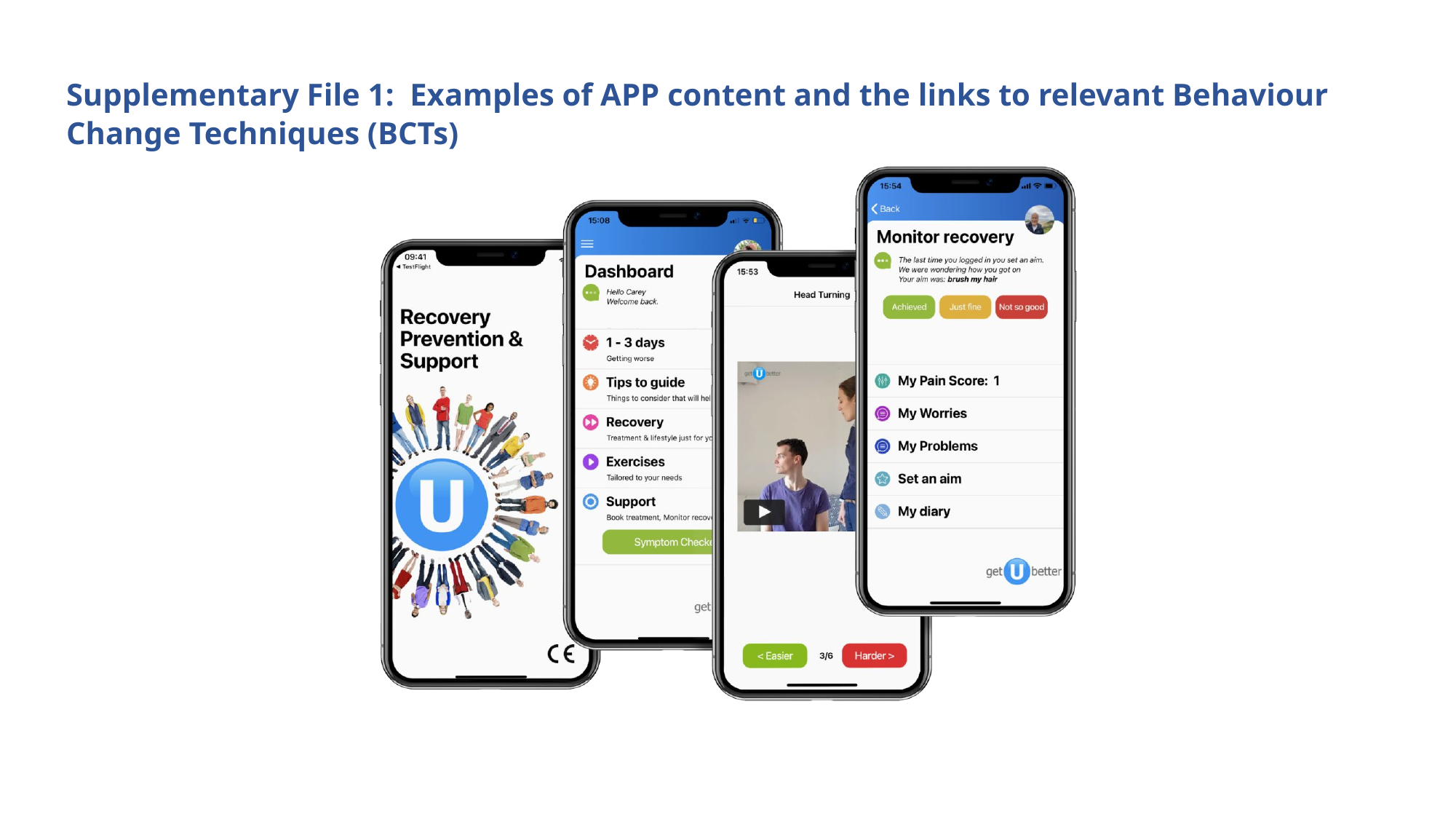

Supplementary File 1: Examples of APP content and the links to relevant Behaviour Change Techniques (BCTs)

## Slide 2
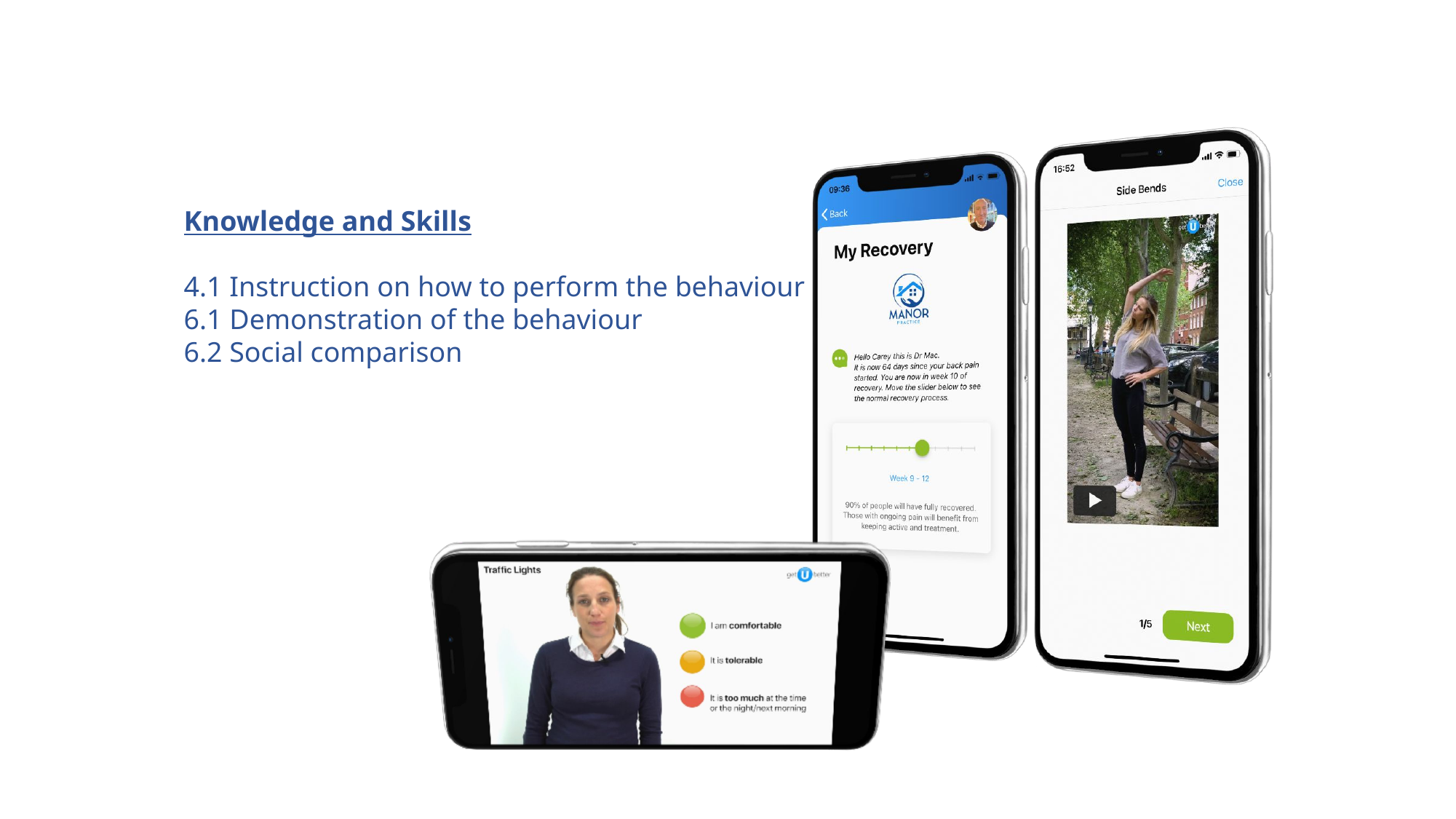

Knowledge and Skills
4.1 Instruction on how to perform the behaviour
6.1 Demonstration of the behaviour
6.2 Social comparison

## Slide 3
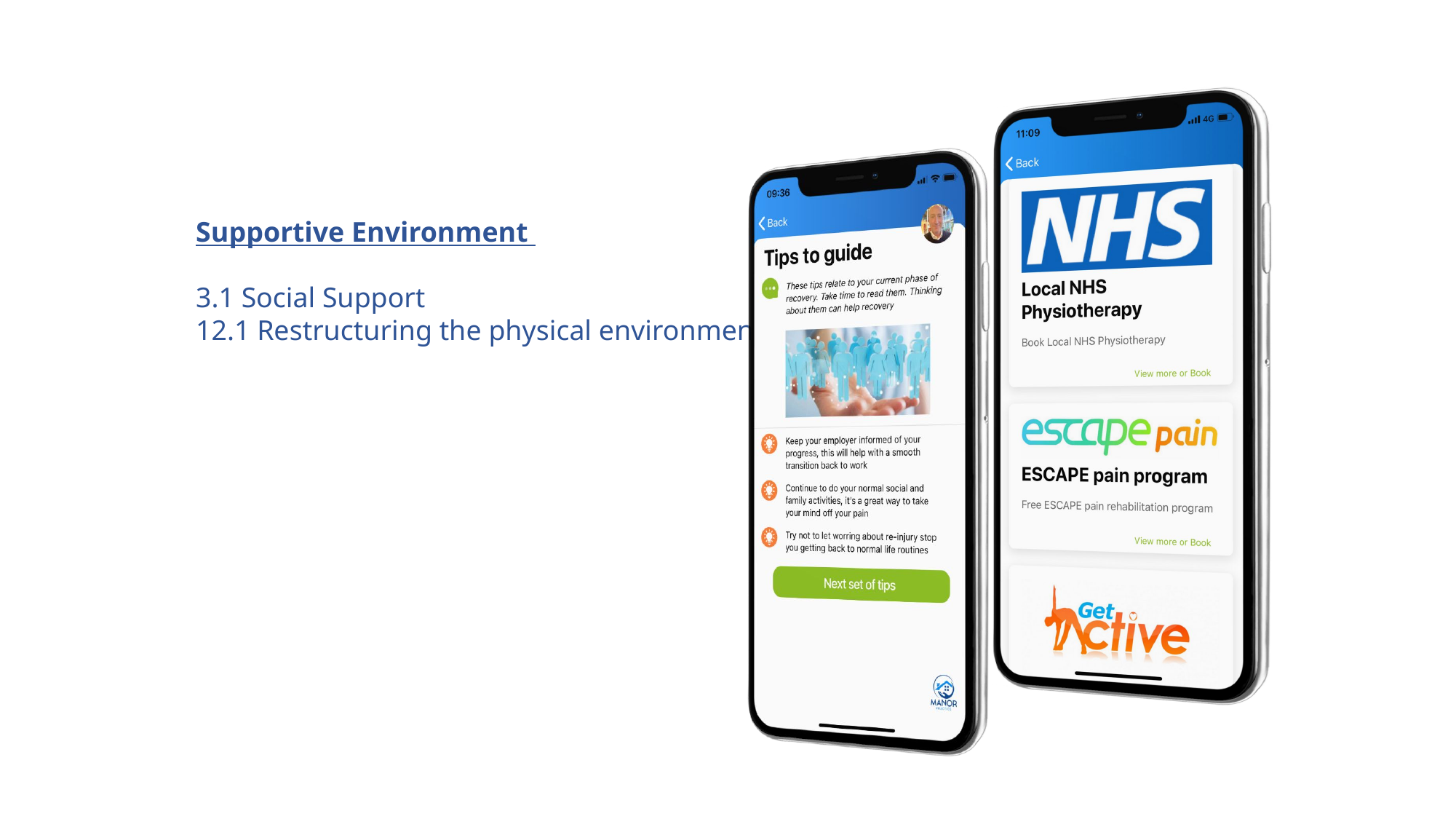

Supportive Environment
3.1 Social Support
12.1 Restructuring the physical environment

## Slide 4
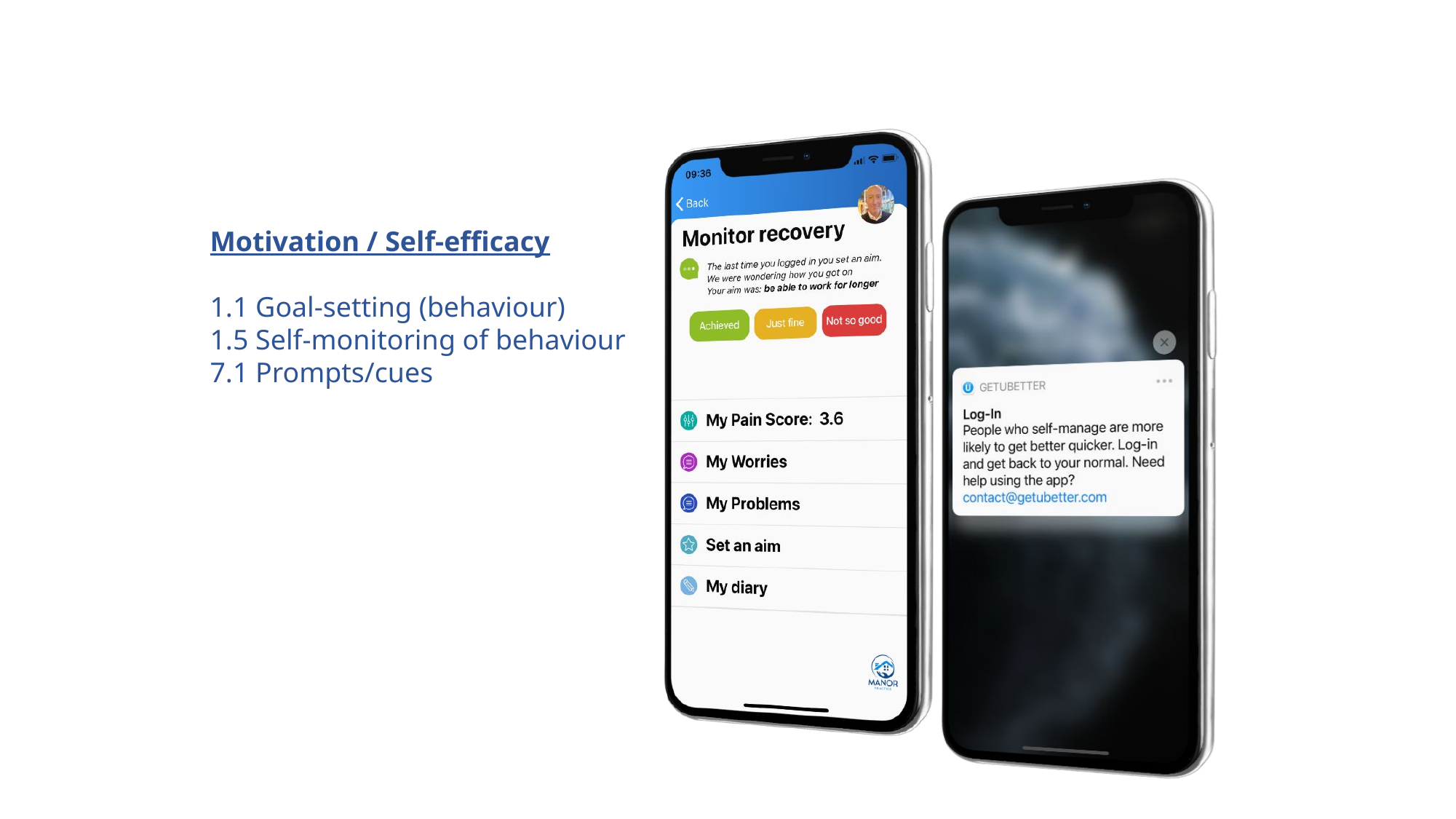

Motivation / Self-efficacy
1.1 Goal-setting (behaviour)
1.5 Self-monitoring of behaviour
7.1 Prompts/cues
