# Supplementary material for: A Tailored App for the Self-management of Musculoskeletal Conditions: Evidencing a Logic Model of Behavior Change
Source: JMIR Form Res. 2022 Mar 8;6(3):e32669. doi: 10.2196/32669 (PMC8941434; doi:10.2196/32669)
Supplement: Multimedia Appendix 2 [file formative_v6i3e32669_app2.docx]

## getUBetter – Linking COM-B components to intervention functions and policy categories from the BCW

| **COM-B model component** | **Definition/Description** | **Relevant COM-B sub-classification (see page 117, Michie et al 2014)** | **Intervention function (BCW)** | **LINK TO getUBetter AIMS/OBJECTIVES** | **Relevant Policy categories (BCW) that could deliver intervention functions (BCW)** |
| --- | --- | --- | --- | --- | --- |
| Physical **CAPABILITY** | Physical skill, strength or stamina | SKILL | **TRAIN** in **physical skills** required for desired behaviour | 1B | Guidelines  Fiscal measures  Regulation  Service provision |
| Psych. **CAPABILITY** | Knowledge or psych skills, strength or stamina to engage in the necessary mental processes | KNOWLEDGE | **EDUCATE** **about ways** of enacting the desired behaviour | 1A | Communication/marketing  Guidelines  Regulation  Service provision |
|  |  | SKILL | **TRAIN** in **cognitive/social skills** required for desired behaviour | 1B | Guidelines  Fiscal measures  Regulation  Service provision |
|  |  | STRENGTH | **TRAIN /ENABLEMENT –** develop **mental/physical strength** required to carry our desired behaviour | 1B | Guidelines  Fiscal measures  Regulation  Environmental/social planning  Service provision |
|  |  | STAMINA/ENDURANCE | **TRAIN/ENABLEMENT develop endurance** required for desired behaviour | 3C | Guidelines  Fiscal measures  Regulation  Environmental/social planning  Service provision |
| Physical **OPPORTUNITY** | Opportunity afforded by the environment involving time, resources, locations, cues, physical ‘affordance’ | TIME | **TRAIN/RESTRUCTURE** the **environment** to reduce time demand or competing time demands for desired behaviour | 2D | Guidelines  Fiscal measures  Regulation  Environmental/social planning  Service provision |
|  |  | RESOURCES | **RESTRUCTURE THE ENVIRONMENT** to increase social support and cultural norms for desired behaviour | 2B, 2C | Guidelines  Fiscal measures  Regulation  Environmental/social planning |
|  |  | LOCATION/PHYSICAL BARRIERS | **TRAIN or RESTRUCTURE THE ENVIRONMENT** to provide cues and prompts for desired behaviour | 3D | Guidelines  Fiscal measures  Regulation  Environmental/social planning  Service provision |
| Social **OPPORTUNITY** | Opportunity afforded by interpersonal influences, social cues and cultural norms that influence the way that we think about things, eg the words and concepts that make up our language | INTERPERSONAL INFLUENCES/CULTURAL EXPECTATIONS | **RESTRUCTURE THE SOCIAL ENVIRONMENT** or use **MODELLING** to shape people’s way of thinking | 2C, 1B | Communication/marketing  Guidelines  Fiscal measures  Regulation  Environmental/social planning  Service provision |
| Reflective **MOTIVATION** | Reflective processes involving plans (self-conscious intentions) and evaluations (beliefs about what is good and bad) | PLANS | **EDUCATE/TRAIN** to form clearer personal action plans/train to remember and apply rules when needed | 3C | Communication/marketing  Guidelines  Fiscal measures  Regulation  Service provision |
|  |  | EVALUATIONS | **EDUCATE/PERSUADE** to create more positive beliefs about desired behaviours | 3B | Communication/marketing  Guidelines  Regulation  Service provision |
| Automatic **MOTIVATION** | Automatic processes involving emotional reactions, desires (wants and needs), impulses, inhibitions, drive states and reflex responses. | MOTIVES | **PERSUADE/MODEL/ENABLE** to feel positively about desired behaviour | 3A | Communication/marketing  Guidelines  Fiscal measures  Regulation  Environmental/social planning  Service provision |
|  |  | IMPULSES/INHIBITION | **TRAIN/ENABLE** to strengthen habitual engagement in desired behaviour | 3C | Guidelines  Fiscal measures  Regulation  Environmental/social planning  Service provision |
|  |  | RESPONSES | **MODEL** desired behaviour to induce automatic induction | 1B | Communication/marketing  Service provision |
